# Heterozygous loss-of-function variants in *DOCK4* cause neurodevelopmental delay and microcephaly

**DOI:** 10.1007/s00439-024-02655-4

**Published:** 2024-03-25

**Authors:** Charlotte Herbst, Viktoria Bothe, Meret Wegler, Susanne Axer-Schaefer, Séverine Audebert-Bellanger, Jozef Gecz, Benjamin Cogne, Hagit Baris Feldman, Anselm H. C. Horn, Anna C. E. Hurst, Melissa A. Kelly, Michael C. Kruer, Alina Kurolap, Annie Laquerriere, Megan Li, Paul R. Mark, Markus Morawski, Mathilde Nizon, Tomi Pastinen, Tilman Polster, Pascale Saugier-Veber, Jang SeSong, Heinrich Sticht, Jens T. Stieler, Isabelle Thifffault, Clare L. van Eyk, Pascale Marcorelles, Myriam Vezain-Mouchard, Rami Abou Jamra, Henry Oppermann

**Affiliations:** 1 Institute of Human Genetics, University of Leipzig Medical Center, 04103 Leipzig, Germany; 2 Department of Epileptology, Krankenhaus Mara Bethel Epilepsy Center Medical School OWL, Bielefeld University, Campus Bethel, Bielefeld, Germany; 3 Department of Genetics, CHU Brest, 29000 Brest, France; 4 Adelaide Medical School and Robinson Research Institute, The University of Adelaide, Adelaide, SA Australia; 5 Service de Génétique Médicale, CHU Nantes, 44000 Nantes, France; 6 l’institut du Thorax, Nantes Université, CHU Nantes, CNRS, INSERM, 44000 Nantes, France; 7 The Genetics Institute and Genomics Center, Tel Aviv Sourasky Medical Center, Tel Aviv, Israel; 8 Sackler Faculty of Medicine, Tel Aviv University, Tel Aviv, Israel; 9 Institute of Biochemistry, Friedrich-Alexander-Universität Erlangen-Nürnberg, Erlangen, Germany; 10 Erlangen National High Performance Computing Center, Friedrich-Alexander-Universität Erlangen-Nürnberg, Erlangen, Germany; 11 Department of Genetics, University of Alabama at Birmingham, Birmingham, AL USA; 12 HudsonAlpha Clinical Services Lab, HudsonAlpha Institute for Biotechnology, Huntsville, AL USA; 13 Barrow Neurological Institute, Phoenix Children’s Hospital University of Arizona College of Medicine, Phoenix, USA; 14 Department of Anatomy, Inserm U1245 and CHU Rouen, Univ Rouen Normandie, 76000 Rouen, France; 15 Invitae Corp, San Francisco, CA USA; 16 Division of Medical Genetics, Helen DeVos Children’s Hospital, Corewell Health, Grand Rapids, MI USA; 17 Center of Neuropathology and Brain Research, Medical Faculty, Paul Flechsig Institute, University of Leipzig, Leipzig, Germany; 18 Genomic Medicine Center, Children’s Mercy Hospital, Kansas City, USA; 19 University of Missouri Kansas City School of Medicine, Kansas City, USA; 20 Department of Genetics and Reference Center for Developmental Disorders, Inserm U1245 and CHU Rouen, Univ Rouen Normandie, 76000 Rouen, France; 21 Genomic Medicine Institute, Seoul National University, Seoul, Republic of Korea; 22 Department of Anatomy, CHU Brest, 29000 Brest, France

## Abstract

Neurons form the basic anatomical and functional structure of the nervous system, and defects in neuronal differentiation or formation of neurites are associated with various psychiatric and neurodevelopmental disorders. Dynamic changes in the cytoskeleton are essential for this process, which is, inter alia, controlled by the dedicator of cytokinesis 4 (*DOCK4*) through the activation of *RAC1*. Here, we clinically describe 7 individuals (6 males and one female) with variants in *DOCK4* and overlapping phenotype of mild to severe global developmental delay. Additional symptoms include coordination or gait abnormalities, microcephaly, nonspecific brain malformations, hypotonia and seizures. Four individuals carry missense variants (three of them detected de novo) and three individuals carry null variants (two of them maternally inherited). Molecular modeling of the heterozygous missense variants suggests that the majority of them affect the globular structure of DOCK4. In vitro functional expression studies in transfected Neuro-2A cells showed that all missense variants impaired neurite outgrowth. Furthermore, *Dock4* knockout Neuro-2A cells also exhibited defects in promoting neurite outgrowth. Our results, including clinical, molecular and functional data, suggest that loss-of-function variants in *DOCK4* probable cause a variable spectrum of a novel neurodevelopmental disorder with microcephaly.

**Supplementary Information:**

The online version contains supplementary material available at 10.1007/s00439-024-02655-4.

## Introduction

Global developmental delay (DD), often leading to intellectual disability (ID), has a prevalence of about 2% and is among the most frequent indications for genetic testing (Sheridan et al. 2013). Despite continuous improvements in sequencing technologies, a genetic cause for DD/ID can only be found in 30 to 50% of routine diagnostic cases (Klau et al. 2022). The high genetic heterogeneity is one reason for the incomplete diagnostic rate. According to the SysNDD (Kochinke et al. 2016) database, pathogenic variants in 1780 (gene statistics from October 26, 2023) different genes are currently established as causes of diverse neurodevelopmental disorders (NDD). Although the rate of discovery of new rare disease–gene associations has declined in recent years (Ehrhart et al. 2021), several disease-causing genes remain undiscovered, as demonstrated by a recent analysis of the X chromosome (Leitão et al. 2022).

Neurons form the basic anatomical and functional structures of the nervous system and consist of two structural components: a cell soma and neurites that include a single axon and dendrites. The length of the neurons varies depending on their function and can be 10,000 to 15,000 times the diameter of the neuronal cell body (Larsen et al. 2016). The formation and function of dendrites and neurites are highly dependent on dynamic changes in the cytoskeleton that are regulated by small GTPases (Nimchinsky et al. 2002). Disease-causing variants in genes that are involved in this process, such as *RHOA* (MIM: #618,727), *CDC42* (MIM: #616,737) and *RAC1* (MIM: #617,751), are associated with a NDD. In addition, dysregulation of guanine nucleotide exchange factors that regulate RAC1 activity is also associated with DD/ID. For example, bi-allelic null variants in *DOCK6* (MIM: #614,219) and *DOCK7* (MIM: #615,859), members of the dedicator of cytokinesis (DOCK) family, are a cause for the Adams–Oliver syndrome (Sukalo et al. 2015) and epileptic encephalopathy (Perrault et al. 2014), respectively. Furthermore, Wiltrout et al*.* reported three individuals with DD, hypotonia, coordination or gait abnormalities and bi-allelic variants in *DOCK3* (MIM: #618,292) (Wiltrout et al. 2019). Together with *DOCK3*, *DOCK4* belongs to the DOCKB subfamily and has four conserved domains: the N-terminal located src Homology-3 (SH3) domain, a proline-rich C terminus and the dock homology regions (DHR) 1 and 2; the latter is responsible for binding and activation of *RAC1* (Shi 2013). The *DOCK4*-dependent *RAC1* activation has been reported as important for neurite differentiation (Xiao et al. 2013) and interaction with the proline-rich C terminus with cortactin for dendritic spine formation (Ueda et al. 2013).

In this study, we report seven individuals with an overlapping phenotype of mild to severe DD/ID, harboring different variants in *DOCK4*. We used structural in silico modeling and a neurite outgrowth assays with Neuro-2A cells that were transiently transfected with an overexpression plasmid for *DOCK4* or after CRISPR-Cas9-mediated *Dock4* knockout to assess possible impact of the variants.

## Materials and methods

### Research cohort and identification of variants

By using match making platforms (Sobreira et al. 2015), personal communication and a literature review, nine different families and 17 individuals from the literature (for further details see Supplemental Information) with potential causative *DOCK4* variants were assessed. Genotypic and detailed phenotypic information was obtained from referring collaborators for eight individuals, seven of whom were included into the clinical description, using a standardized questionnaire assessing family history, clinical history, genetic testing, variant details, EEG, brain imaging and medication (see Table S1). All individuals underwent single or trio exome or genome sequencing using local protocols. Maternity and paternity were confirmed for all individuals, and Sanger sequencing was used for segregation analysis where appropriate. As no causative variants in a known disease gene were identified that explains DD/ID of the individuals, the data were examined in a scientific approach, including parental sequence data if available. All variants were prioritized considering allele frequency in gnomAD v4.0.0 (Karczewski et al. 2020) below 1%, impact on protein function via different in silico programs (CADD (Rentzsch et al. 2021), REVEL (Ioannidis et al. 2016), MutPred2 (Pejaver et al. 2020) and Metadome (Wiel et al. 2019)) and involvement of candidate genes in neuronal processes. Apart from the *DOCK4* variants observed, none of the individuals described had other remarkable findings nor other candidates that likely explain the phenotype. All variants in *DOCK4* are described with regard to the human reference genome version GRCh38 and to the transcript NM_014705.4 and have been classified according to the criteria of the American College of Medical Genetics (ACMG) (Richards et al. 2015) and the recommendations of the Sequence Variant Interpretation Working Group (ClinGen).

### *DOCK4* expression plasmids

Expression plasmids were generated as described previously (Rahimi et al. 2022), by using full-length human *DOCK4* open reading frame (ORF; GeneBank: BC117689; MHS6278-211,690,438) that was obtained from Horizon Discovery BioSciences (Cambridge, UK). Fragments of the *DOCK4* were amplified by PCR (TableS2) from *DOCK4* ORF by using Q5 High Fidelity DNA Polymerase (New England Biolabs, Frankfurt am Main, Germany) and were assembled together with the pcDNA3.1_mRFP Vector by using the NEBuilder HiFi DNA Assembly Cloning kit (New England Biolabs) according to the manufactures recommendation to obtain a plasmid that express a *DOCK4* (GeneBank sequence: NM_014705) mRFP fusion protein. Mutagenesis was performed by using the Q5 Site-Directed Mutagenesis Kit (New England Biolabs) according to the manufacturer’s recommendation. All sequences were verified by Sanger sequencing.

### Cell culture and transfection

Transfection was performed as described previously (Rahimi et al. 2022). Briefly, Neuro-2A cells (RRID:CVCL_0470) were originally obtained from the DSMZ (German Collection of Microorganisms and Cell Cultures, Braunschweig, Germany) and cultured in T‐75 culture flasks (Sarstedt AG & Co.) in DMEM/25 mM glucose, without pyruvate (Thermo Fisher Scientific), supplemented with 10% fetal bovine serum (FBS superior, Biochrom), 2 mM l‐alanyl‐l‐glutamine and antibiotics (Thermo Fisher Scientific) at 37 °C and 5% CO2 in humidified air in an incubator. For transfection of plasmids, Neuro-2A cells were seeded in 12-well plates and transfected with 6 μL JetPEI (Polyplus, Illkrich, France) and 2 μg of DNA encoding wild-type or mutated variants of *DOCK4*-mRFP.

### Generation of *Dock4* KO Neuro-2A cells with CRISPR/Cas9

*Dock4* KO cells were generated by using the Alt-R CRISPR-Cas9 system (Integrated DNA Technologies, Leuven, Belgium) with Lipofectamine RNAiMAX (Thermo Fisher Scientific) according the manufactures recommendation. For the experiments, predesigned guide RNA sequences were used (ko1: 5’-UCAUGAUGUGCCACAGGCGA-3’, ko2: 5’- ACAUUACUGCCCGACUGGAU-3’). Control cells were generated by using the Alt-R CRISPR-Cas9 Mouse Control Kit (Integrated DNA Technologies). 24 h post-transfection, cells were collected and seeded at a density of 0.8 cells per 100 µl into 96-well plates. After 10–12 days of culture, wells containing only one colony were split into two wells of a 96-well plate and positive clones confirmed by DNA sequencing were further propagated. Knockout clones were finally confirmed by western blotting.

### Western blotting

Western blotting was performed as described previously (Oppermann et al. 2019). Briefly, 30 µg of protein was separated on a 4–20% SDS-PAGE (sodium dodecyl sulfate–polyacrylamide gel electrophoresis) gradient gel (Bio-Rad, Munich, Germany) and was transferred to a PVDF (polyvinylidene difluoride) membranes (Low Fluorescence Membrane Opti Blot, Abcam, Cambridge, UK). The primary antibodies used were: mouse anti-DOCK4 (Santa Cruz Biotechnology, Heiderlberg, Germany #sc-100718 1:500), rabbit anti-GAPDH antibody (Cell Signaling; #2118 1:5000) and mouse anti-ACTB (Novus Biologicals #NB600-501, 1:5000). The secondary antibodies employed (red fluorescent IRDye 680RD goat anti-mouse and green fluorescent IRDye 800CW goat anti-rabbit; both diluted 1:10,000 in TBST) were purchased from LI-COR (LI-COR Biosciences, Lincoln, USA). Membranes were scanned using an Odyssey Imaging System (LI-COR, Bad Homburg, Germany), and band intensities were determined by the Image Studio 5 software (LI-COR).

### Neurite outgrowth assay

After transfection, cells were collected and subjected to FACS sorting by using a BD FACSAria II SORP (BD Biosciences, Heidelberg, Germany). 15,000 of mRFP-selected cells were seeded per well into a 96-well plate, and culture media was exchanged after 4 h to Neurobasal A medium containing GlutaMAX, antibiotics and 2% B27 supplement (Thermo Fisher Scientific). For *Dock4* KO cells, which were not transfected, 7500 cells per well in a 96-well plate were seeded. Afterward cells were cultured (37 °C and 5% CO2 in humidified air) and monitored in a Celldiscoverer 7 (Zeiss, Oberkochen, Germany). Neurite length was determined after 36 h in differentiation media by using NeuronJ (Meijering et al. 2004) implemented in ImageJ. The result of each assay per group was confirmed by at least two individual experiments, and representative data are shown.

### Molecular modeling

The structural analysis of *DOCK4* was based on a model available in the AlphaFold Protein Structure Database at: https://alphafold.ebi.ac.uk/entry/Q8N1I0 (Jumper et al. 2021; Varadi et al. 2022). The binding sites of ELMO and RAC1 were deduced from the ELMO1-DOCK5-Rac1 complex (PDB: 7DPA (Kukimoto-Niino et al. 2021)). Mutations were modeled with SWISS-MODEL (Guex and Peitsch 1997), and VMD (Humphrey et al. 1996) was used for structure analysis and visualization. Sequence-based predictions for those variants located in nonglobular regions were performed using the ELM server (Kumar et al. 2022). Experimentally confirmed phosphorylation sites were retrieved from PhosphoSitePlus (Hornbeck et al. 2015).

### Statistical analysis

Statistical analysis was carried out using SPSS (IBM, Armonk, USA; version: 24.0.0.2 64-bit). We used a one-way ANOVA with the Games–Howell post hoc test for multiple comparisons. If not stated otherwise, data are presented as mean ± SEM. A *p* value < 0.05 was presumed to be statistically significant.

## Results

### Clinical description

In this study, we report on seven individuals with heterozygous rare variants in *DOCK4*. A summary of the clinical symptoms of each individual is presented in Table 1, and a full clinical description is given in Table S1 and in the Supplemental Information.

**Table 1:** Clinical spectrum of the *DOCK4*-related disorder

Individual #	Variants	Inheritance	Sex	Age*	DD	ID	Microcephaly	Coordination or gait abnormalities	Other
1	c.2945C > T; p.Thr982Ile	de novo	M	14y 8 m	Y, mod	Y, mod-sev		Tremor, ataxia	Seizures
2	c.3131 T > C; p.Met1044Thr and c.758C > T; p.Pro253Leu	de novo and paternal	M	5 y	Y, mild	Y, mild-mod	Y	Ataxia, spasticity	Brain malformation, facial dysmorphism, ophthalmologic abnormalities, hypotonia
3	c.3200 T > C: p.Ile1067Thr	de novo	M	5 y	Y, mild	N	N	Spastic hemiplegia, dystonia	Brain malformation
4	c.5886G > T; p.Lys1962Asn	unknown	M	12 y	Y, sev	Y, mod	N	Dystonia	Brain malformation, facial dysmorphism, ASD, seizures, ophthalmologic abnormalities, hypotonia
5	c.892C > T; p.Arg298*	maternal	M	6 y	Y, mild	Y, mild	Y	N	Facial dysmorphism, ASD
6	c.2770C > T; p.Arg924*	unknown (not maternal)	F	20 y	Y, mild	N		N	Attention disorder, anxiety, global learning difficulties, ophthalmologic abnormalities
7	c.3910del; p.Asp1304Thrfs*27	maternal	M	5 y 6 m	Y, mod	N/A	N	Cerebral palsy, increased axial tone	Brain malformation, facial dysmorphism, seizures, ophthalmologic abnormalities, hypotonia

y = year, ID = intellectual disability, DD = development delay, ASD = autism spectrum disorder N/A = not available, age* = age at last examination, M = male, F = female; c code is based on the transcript: NM_014705.4

**Fig. 1:**
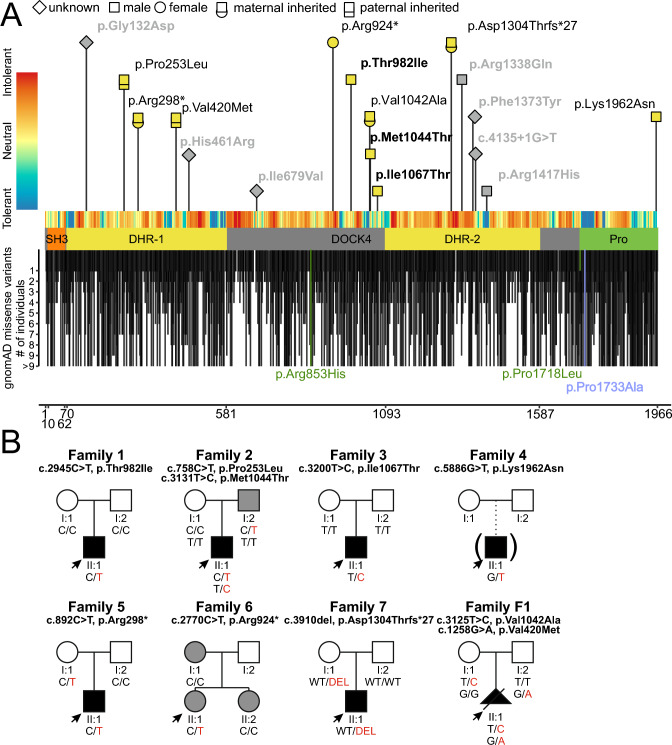
Variants in *DOCK4* and pedigrees. **A** Location of missense and null variants in *DOCK4* with respect to the *DOCK4* domain structure (GenBank: NM_014705.4). The x-axis represents the corresponding *DOCK4* amino acid position of *DOCK4*. Variants reported in this study are labeled with the corresponding p-code; females are indicated as circles and males as squares. Indicated in bold are confirmed de novo variants. De novo variants that were identified in individuals with global development delay from other studies with a lacking detailed phenotypic description or inconclusive evidence are indicated in gray (see also Table S1). Missense variants in gnomAD with allele count are shown below the protein scheme. Positive controls (used for neurite outgrowth assay; p.Arg853His, p.Pro1718Leu) are labeled in green and the negative control (p.Pro1733Ala) is labeled in blue. The tolerance landscape from MetaDome is shown color-coded above the protein scheme. **B** Family pedigrees. Individuals with *DOCK4*-related disorder are indicated by filled black shapes with an arrow pointing to the individual. Open shapes represent unaffected individuals. Squares represent males, circles represent females, and triangles with a diagonal line represents termination of pregnancy. Gray-colored shapes indicate individuals with comparable milder symptoms. Of note, the phenotype of the father (I:2) of individual II:1 (family 6) is unknown. A = Generation number; B = number of that individual in that generation. Segregation results for all individuals tested are indicated with either red (presence of the *DOCK4* variant) and/or black (presence of the reference allele). Abbreviations: SH3: N-terminal Src homology 3; DHR: dock homology region; *DOCK4*: dedicator of cytokinesis protein 4; Pro: proline-rich C-terminal end; WT: wild type

All individuals presented with DD of variable severity, ranging between mild (individuals 2, 3, 5 and 6), moderate (individuals 1 and 7) and severe (individual 4). The developmental course of individual 1 differs from the others, with normal developmental milestones until the first epileptic seizure at 4 years of age, followed by moderate to severe developmental delay. Cognitive impairment was highly variable, ranging from learning difficulties with no ID (individuals 3 and 6) to mild (individuals 2 and 5) and moderate ID (individuals 1 and 4). Behavioral abnormalities were less common, with a diagnosis of autism or autistic behavior in individuals 4 and 5, and attention disorder and anxiety in individual 6. Of note, the father of individual 2, who transmitted one variant in *DOCK4* (Fig. 1B), has a bipolar disorder, attention deficit hyperactivity disorder, anxiety and dyslexia.

Different coordination or gait abnormalities were reported in five out of seven individuals. In detail, individual 1 had ataxia and tremor, individual 2 had ataxia and spasticity, individual 3 had dystonia and spastic hemiplegia, individual 4 had dystonia and individual 7 had cerebral palsy. Other neurological symptoms were muscular hypotonia (individuals 2, 4 and 7) and different types of seizures. Individual 1 had focal to bilateral tonic–clonic seizures, and individual 7 had an abnormal EEG that is consistent with a focal onset seizure.

Six individuals underwent MRI and four had nonspecific brain abnormalities. Individual 2 had mild cerebellar ectopia, and individual 3 had a complex volume loss in the left periventricular region, extending into basal ganglia and part of brainstem, with thinning of the corpus callosum and suggestion of incomplete myelination in surrounding areas. Individual 4 had mild diffuse cerebral atrophy and minimal white matter gliosis and individual 7 bilateral cerebral hemispheric white matter volume loss and gliosis.

Additional findings include microcephaly in individuals 2 (OFC: 43.3 cm; Z-score: -3.84) and 7 (OFC: 48 cm; Z-score: -2). Although minor facial dysmorphisms have been reported for four individuals (individuals 2, 4, 5 and 7), there was no apparent shared facial gestalt (Table S1).

It should be noted that we also identified two compound heterozygous missense variants in a fetus with microcephaly, rhombencephalosynapsis and microlissencephaly (Table S1). As the pregnancy was aborted, no further clinical information is available. As the assumed inheritance is not consistent with the rest of the cohort, we have not included this case in the clinical description. Nonetheless, this fetus had the most severe structural anomalies of the cerebellum and brainstem (for details see Supplemental Information).

### Genetic findings

In total, we identified ten clinical relevant variants in *DOCK4*: seven missense and three null variants (Fig. 1A). In three individuals, we detected a de novo missense variant. Segregation analysis of individual 4 was not possible because he was adopted. Two null variants were maternally inherited, and the inheritance of the null variant of individual 6 is unknown (Fig. 1B). The missense variants p.Thr982Ile, p.Met1044Thr and p.Ile1067Thr were absent from gnomAD. The variants p.Lys1962Asn and p.Val1042Ala were detected in gnomAD once, and the two inherited missense variants p.Pro253Leu and p.Val420Met (detected compound heterozygous with the variant p.Val1042Ala) were observed with an allele frequency of 0.061% and 0.00186% in gnomAD, respectively. The inherited null variants were also detected in gnomAD with a very low frequency (p.Arg298*: 0.00014%; p.Arg924*: 0.000136%; p.Asp1304Thrfs*27: 0.0001%). It should be noted that the paternally inherited variant p.Pro253Leu was detected together with the de novo variant p.Met1044Thr in individual 2. Unfortunately, phasing of the de novo variant was not possible, but it is more likely that this variant is also located on the paternal allele (i.e., *in cis*) (Jónsson et al. 2017). For the three identified null variants, it can be assumed that the mRNA is degraded by nonsense-mediated mRNA decay (Lindeboom et al. 2016) causing haploinsuffuciency. Based on gnomAD (Karczewski et al. 2020), *DOCK4* has a reduced amount of missense and null variants compared to its size of 1,966 amino acids (pLI = 1, Z score = 1.67) in a population without severe, early-onset phenotypes, indicating a mild constraint. The missense variants from individuals 2 and 3 are predicted to be deleterious by CADD and MutPred2 (Table S1). The missense variants are located in the proline-rich C-terminal end, in the DHR-1 and proximal of the DHR-2 domain (Fig. 1). A clustering of disease-causing missense variants in a specific region of *DOCK4* was not observed. Only several small parts of *DOCK4* seem to be highly depleted of missense variants in the general population (Fig. 1A).

### *DOCK4* missense variants affect the globular structure of DOCK4

**Fig. 2:**
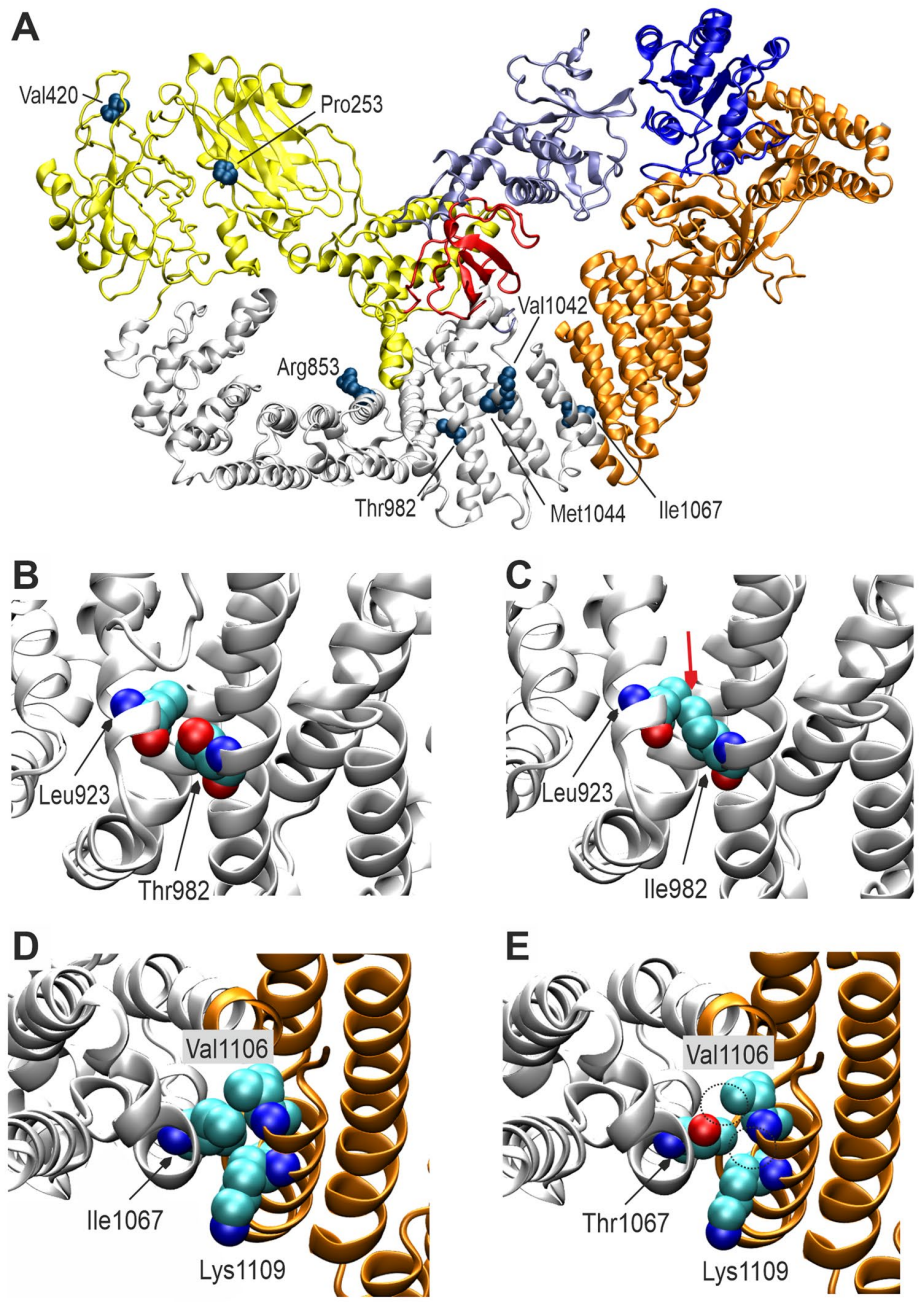
Structural analysis of the *DOCK4* variants. **A** Model of the DOCK4 globular region (residues 1-1587) indicating the sites of the variants of interest in space-filled presentation. The SH3, DHR-1, DOCK4 and DHR-2 domain are colored in red, yellow, white and orange, respectively. The interaction partners ELMO and Rac-1 are shown in violet and blue. **B**, **C** Vicinity of residue 982 in the **B** wild-type and **C** p.Thr982Ile variant. In this variant, the longer isoleucine sidechain forms steric clashes (red arrow) with Leu923. **D** Interactions of the Ile1067 sidechain with Val1106 and Lys1109. **E** In the p.Ile1067Thr variant, these interactions cannot be formed by the shorter threonine sidechain (sites of missing interactions marked as dotted circles)

To better understand the impact of the missense variants in *DOCK4*, we performed a structural analysis. As there is no experimental structure of DOCK4 available to date, we used a model generated by AlphaFold-2 for the structural interpretation of the variants. Inspection of the AlphaFold scores indicates a reliable model for residues 1–1587, whereas the low confidence scores for residues 1588–1966 suggest that the C terminus is disordered and lacks a defined three-dimensional structure. Structural modeling of the variants in the globular domain reveals that all of them cause a destabilization of the DOCK4 fold. Depending on the molecular origin of destabilization, the variants can roughly be divided into two categories: introduction of steric clashes and loss of hydrophobic interactions. For each category, the corresponding variants are described below, and a representative variant is shown in Fig. 2. (i) Introduction of steric clashes: The p.Thr982Ile exchange induces steric clashes with Leu923 due to the longer isoleucine sidechain present in the variant (Fig. 2B, C). Similar steric problems emerge from the longer methionine sidechain in the p.Val420Met and the β-branched threonine sidechain in the p.Met1044Thr variant. (ii) Loss of hydrophobic interactions: In the p.Ile1067Thr variant, the isoleucine is replaced by a shorter threonine. This results in a loss of hydrophobic interactions with Val1106 and Lys1109 (Fig. 2D, E). A similar loss of hydrophobic interactions is observed for the p.Pro253Leu and p.Val1042Ala variants.

The variant p.Lys1962Asn is located in the nonglobular C terminus of DOCK4. Such regions may harbor linear motifs that mediate posttranslational modification and/or protein–protein interactions (Kumar et al. 2022). Although the exact role of individual amino acids within this motif has not been experimentally addressed to date, the DOCK4 C terminus (^1962^KVSQL^1966^) matches the [RK]-x-[ST] consensus pattern present in a class of known PDZ ligands (Tonikian et al. 2008). This observation suggests that replacement of the positively charged lysine by an uncharged asparagine in the p.Lys1962Asn variant leads to a decreased affinity of DOCK4 for harmonin and/or other PDZ domain-containing proteins. In contrast, no functional role can be assigned to Pro1733, suggesting that the p.Pro1733Ala exchange (rs150569245, detected with a frequency of 1.163% in gnomAD) does not critically affect DOCK4 function.

### Missense and null variants in *DOCK4* lack neurite outgrowth abilities in Neuro-2A cells

**Fig. 3:**
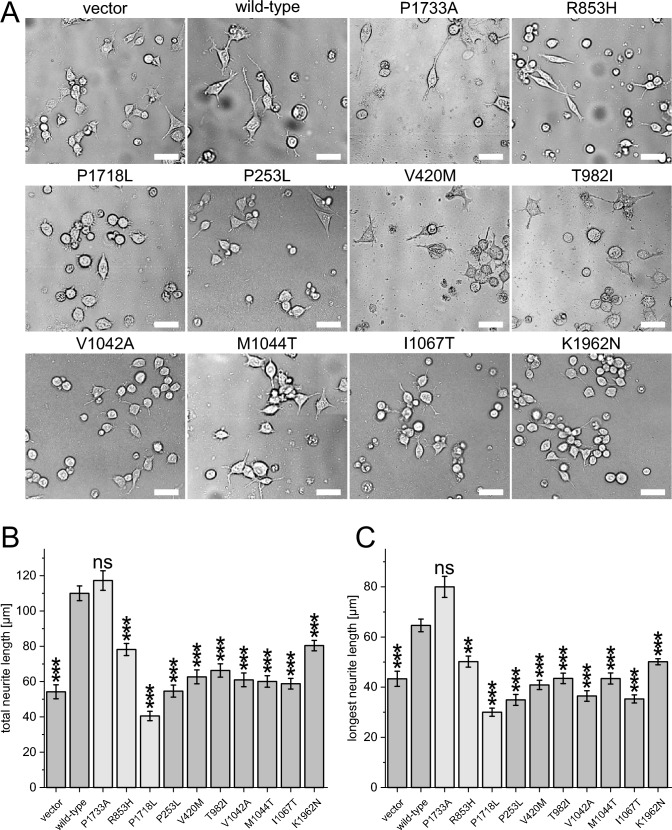
De novo and inherited missense variants in *DOCK4* affect neurite outgrowth in Neuro-2A cells. **A** Neuro-2A cells were transfected with plasmids expressing wild-type *DOCK4*, the negative control p.Pro1733Ala, the positive controls p.Arg853His and p.Pro1718Leu, and the mutant variants p.Pro253Leu, p.Val420Met, p.Thr982Ile, p.Val1042Ala, p.Met1044Thr, p.Ile1067Thr, p.Lys1962Asn or vector. Transfected cells were sorted and collected by flow cytometry and then incubated in Neurobasal A media containing 2% B27 supplement (differentiation media) for 36 h. Scale bar, 50 µm. **B**, **C** Average length of total and average length of the longest neurite were measured. The results of a one-way ANOVA with the Games–Howell post hoc test (each compared to wild type) are indicated as: **: *p* < 0.005; ***: *p* < 0.0005; ns: *p* > 0.05. At least 70 cells/group were analyzed in each experiment. For better readability, the one letter code has been used to describe the variants in this figure

**Fig. 4:**
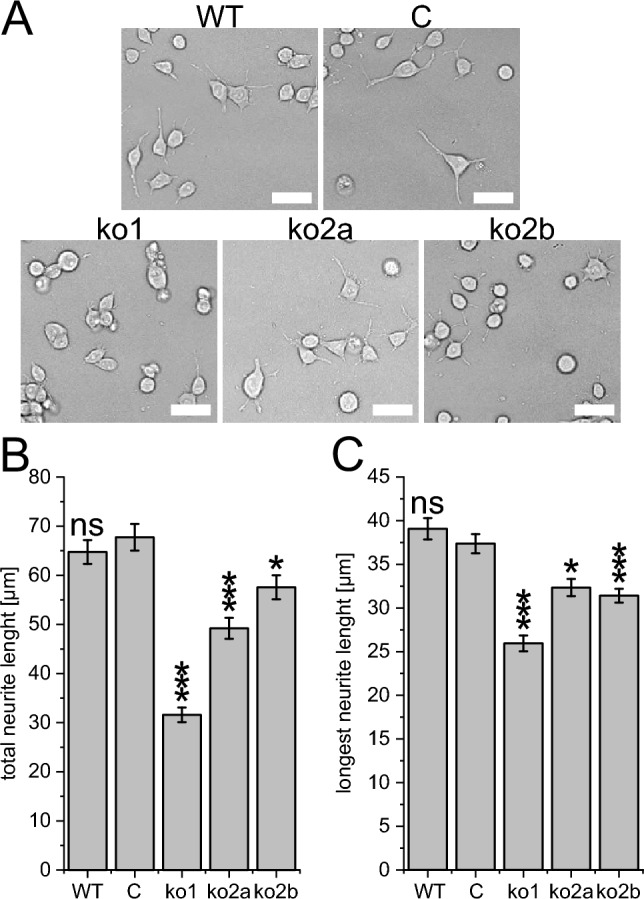
Knockout of *Dock4* in Neuro-2A cells impairs neurite outgrowth. Neuro-2A *Dock4* knockout cells by using the Alt-R CRISPR-Cas9 system utilizing one nonspecific control guide RNA (C) and two different guide RNAs (ko1 and ko2) that were used to generate three knockout clones (ko1, ko2a and ko2b). **A** Neuro-2A cells were incubated in Neurobasal A media containing 2% B27 supplement for 36 h for differentiation. Scale bar, 50 µm. **B** Average length of total and **C** average length of the longest neurite were measured. The results of a one-way ANOVA with the Games–Howell post hoc test (each compared to cells treated with the nonspecific control guide RNA:C) are indicated as: *: *p* < 0.05; ***: *p* < 0.0005; ns: *p* > 0.05. At least 70 cells/group were analyzed in each experiment

As the in silico predictions suggests that all variants affect *DOCK4* function, we tested this assumption experimentally. Huang et al*.* (Huang et al. 2019) demonstrated that the variants p.Arg853Leu and p.Asp946_Lys1966delinsValSer* (described as 945VS) lead to a significantly reduced *DOCK4* dependent activation of Rac1 and Rap1 that resulted in a compromised function in promoting neurite outgrowth in Neuro-2A cells. Therefore, we generated plasmids expressing *DOCK4* and harboring the variants of interest, transfected them into Neuro-2A cells and monitored neurite outgrowth in the presence of differentiation media. In addition, we tested the gnomAD variant p.Pro1733Ala and, as an additional positive control, the variant p.Pro1718Leu that exhibits an impaired function in activating RAP1 (Yajnik et al. 2003). As expected, overexpression of wild-type *DOCK4* promoted neurite formation, as demonstrated by significantly longer neurites compared to cells transfected with the empty vector alone (Fig. 3). Moreover, the gnomAD variant p.Pro1733Ala was indistinguishable from the wild type, supporting its nonpathogenic nature, and the positive controls p.Arg853Leu and p.Pro1718Leu resulted in a significantly reduced neurite outgrowth. More important, all of the variants studied from the individuals also significantly impaired neurite formation in Neuro-2A cells, including the two variants identified in the fetal case. This was demonstrated by both the total and longest neurite length (Fig. 3B, C). Next we experimentally tested whether loss of *DOCK4* also affects neurite outgrowth in Neuro-2A cells. Therefore, we generated Neuro-2A *Dock4* knockout cells by using the Alt-R CRISPR-Cas9 system utilizing two different guide RNAs (ko1 and ko2) and one nonspecific control guide RNA (C: control). *Dock4* knockout was confirmed by DNA sequencing and western blot (Fig. S1). The neurite outgrowth assay demonstrated that the different knockout clones ko1, ko2a and ko2b cells had significant shorter neurites (total and longest neurite length) compared to control and wild-type cells (Fig. 4). Taken together, our experiments demonstrated that null variants and deleterious missense variants lead to impaired function in promoting neurite growth, supporting the association of DD/ID and the variants of the individuals of the present cohort.

## Discussion

In the present study, we describe seven individuals with de novo and inherited heterozygous variants (in total ten different variants, Fig. 1) in *DOCK4*. The affected individuals exhibited a NDD with mild to severe DD, microcephaly and coordination or gait abnormalities. According to ACMG criteria (Richards et al. 2015), three variants can be classified as likely pathogenic and seven as variants of uncertain significance (Table S1). We could demonstrate that null variants (Fig. 4) and the seven missense variants p.Pro253Leu, p.Val420Met, p.Thr982Ile, p.Val1042Ala, p.Met1044Thr, p.Ile1067Thr and p.Lys1962Asn impair neurite formation in Neuro-2A cells. Structural modeling of the variants suggested that all of them cause a destabilization of the DOCK4 fold. Of note, we also tested the variant c.593G > C, p.Ser198Thr (absent from gnomAD) that we identified in a female individual with isolated generalized epilepsy (maternally inherited). A structural modeling of the variant revealed that the sidechain of Ser198 is oriented toward the solvent and does not interact with other amino acids of DOCK4. Therefore, a Ser198Thr exchange is expected to have no critical effect on DOCK4 structure and function. This suggestion could be also confirmed in a neurite outgrowth assay (total neurite length: D4 wild type: 110 ± 4.2µm, p.Ser198Thr: 99.8 ± 3.2 µm; p = 0.77; longest neurite length: D4 wild type: 64.6 ± 2.5µm, p.Ser198Thr: 56.2 ± 1.5 µm; p = 0.183). Therefore, we excluded this individual from the cohort. Moreover, we also identified an individual (Family 8) with the de novo missense variant p.Arg1338Gln and a comparable severe phenotype (severe ID, incomplete head control, incomplete rolling over and no ability to sit at the age of 3 years and 11 months, see Table S1). Interestingly, structural modeling revealed a deleterious effect. Specifically, the side chains of Arg1338 and Asn1411 form a hydrogen bond in the wild-type protein that cannot be formed by the shorter glutamine side chain caused by the p.Arg1338Gln variant. However, the variant was detected in gnomAD with an allele frequency of 0.0003719% (6 counts), which is too high to be considered a monogenetic cause of the severe ID of individual 8. Unfortunately, functional validation of the variant was not possible because multiple attempts to amplify the variant by PCR for mutagenesis failed. As we cannot exclude that the *DOCK4* variant contributes to the symptoms of individual 8, we classified the variant with unknown significance and excluded the individual from the clinical description. It should be noted that the null variants of individuals 5–7, who had a much milder phenotype compared to individual 8, were also detected in gnomAD (Table S1), although the gene seems to be intolerant to null variants (pLI = 1). This observation suggests that pathogenic variants in *DOCK4* could be a cause of relatively mild NDD, as observed in individuals 3, 5 and 6. It can be assumed that such individuals are represented in gnomAD, as individuals with severe pediatric and common adult onset diseases were excluded from the data selection (Karczewski et al. 2020).

The genes of the DOCKB subfamily *DOCK3* and *DOCK4* are important for brain development by promoting neurite and axonal growth and dendritic spine formation (Shi 2013). Interestingly, we could also find a notable overlap of symptoms of the individuals in the present cohort with those harboring bi-allelic disease-causing variants in *DOCK3* (Wiltrout et al. 2019) (Figure S2), supporting *DOCK4* as a NDD gene. In particular, coordination or gait abnormalities, e.g. ataxia, were present in all *DOCK3* individuals and in the majority of individuals (individuals 1, 2, 3, 4 and 7) in the present study.

Based on the detection of rare de novo and inherited missense variants in *DOCK4*, we hypothesized an autosomal dominant mode of inheritance, although we could not rule out an autosomal recessive mode based on the impairment of *DOCK4* function by the variants, i.e., de novo variants would lead to a more severe loss of function compared to inherited variants. With our assay, we could not distinguish between the effects of the de novo and inherited missense variants, which contradicts the hypothesis of such hypomorphic missense variants for *DOCK4*. To further evaluate this hypothesis, we screened for additional *DOCK4* variants in the exome data of individuals carrying a null variant (individuals 5–7). We could detect the coding SNPs p.Pro1733Ala (rs150569245) and p.Val1914Ile (rs12705795) in individual 5 and 7, respectively. Although we have not tested the effect of the SNP p.Val1914Ile, it is unlikely that both SNPs are hypomorphic, as we could demonstrate that the variant p.Pro1733Ala does not affect DOCK4 function (Fig. 3). Therefore, an autosomal dominant mode of inheritance is more likely for the *DOCK4* related NDD. This is also supported by the detection of a heterozygous de novo variant in *DOCK4* in seven individuals with NDD in other studies (see Table S1). Interestingly, *Dock4* knockout in mice leads to early embryonic lethality (Abraham et al. 2015), which is frequently observed in NDD genes with an autosomal dominant mode of inheritance (Cacheiro et al. 2020). In contrast, knockout of the paralogue *Dock3* is not embryonic lethal in mice, but adult animals show a cerebral accumulation of autophagic vacuoles and a disorganization of the axonal cytoskeleton (Chen et al. 2009).

Nevertheless, based on preliminary information from the fetus (F1 in Table S1), an autosomal recessive *DOCK4*-associated disorder may also be possible and will need to be investigated in the future.

In family 6, we detected a null variant in *DOCK4* that did not co-segregate with the symptomatic mother and sister (Fig. 1B, Table S1; a genetic cause for the symptoms of the mother and the sister has not yet been fully investigated). Furthermore, the phenotype of individual 6 is milder (mild DD, no coordination or gait abnormalities, learning difficulties and no ID) and thus different from the rest of the cohort. Although it is possible that null variants in *DOCK4* could be causative for a mild NDD as discussed above, it is unclear whether this variant is causative for the symptoms of individual 6. However, it is not known whether the variant is inherited or whether the father is symptomatic, as there is no contact with the father. More important, all other individuals of the cohort are male, which could indicate a sex-specific expressivity. Pagnamenta et al*.* (2010) described a family with eight individuals (two females and six males) harboring a *DOCK4* truncating deletion (p.Asp946_Lys1966delinsValSer*). The two clinically characterized females had an unremarkable development and were diagnosed with dyslexia. Two of the affected males had DD and were diagnosed with autism. The IQ was in the normal range. Three other males had significant problems in reading and spelling and one male was diagnosed with Asperger disorder. (No developmental milestones were available.) This report indicates an intrafamilial variability of *DOCK4* variants, with males more severely affected than females. However, the two brothers with autism also inherited a deletion that disrupts *CNTNAP5* from their father, who had dyslexia as a child. Although a gene–disease association for *CNTNAP5* has not yet been confirmed, Pagnamenta et al*.* suggested that the *CNTNAP5* deletion contributed to the brothers' autism. Guo et al*.* (2021) investigated autism spectrum disorder like behavior in conditional *Dock4* knockout mice and also observed sex-specific effects. For example, knockout males showed higher anxiety levels and poorer working memory compared to knockout female mice. Noteworthy, overexpression of *Rac1* restored excitatory synaptic transmission and corrected the impaired social behavior of *Dock4* knockout mice. In contrast, there is no enrichment of females (51.5%) in gnomAD with a null variant in *DOCK4* leading to nonsense-mediated decay. Taken together, these findings provide preliminary evidence for sex-specific variable expressivity within autosomal dominant *DOCK4*-related NDD. However, this assumption needs confirmation in a larger cohort.

Evaluating the current evidence for a gene–disease association of *DOCK4* according to the recommendations of ClinGen (Strande et al. 2017), including the experimental evidence of the published cell culture studies (Ueda et al. 1993; Huang et al. 2019), mouse studies (Guo et al. 2021) and the cohort of the present study, would result in supporting evidence of moderate level. However, the application of the ClinGen evaluation criteria is questionable for phenotypes with sex-dependent variable expressivity, such as observed for *DOCK4*, as specific guidelines have not yet been defined for this scenario. To better understand the variable expressivity of *DOCK4*-associated NDD, a larger cohort with detailed segregation information from multiple families would be required.

Our data provide some insight into the underlying pathomechanism. Specifically, our in silico structural modeling suggests a loss-of-function mechanism for the missense variants, which is a comparable mechanism for the null variants. Consistent with this, both *Dock4* knockout and the missense variants investigated resulted in impaired function in promoting neurite outgrowth in Neuro-2A cells. A gain-of-function mechanism of missense variants is unlikely because overexpression of wild-type *DOCK4* results in increased neurite outgrowth capabilities and not the opposite (Fig. 3). Furthermore, loss of *DOCK4* function is compensated by overexpression of the *DOCK4* interacting partner *RAC1*, as demonstrated in vitro (Huang et al. 2019) and in vivo (Guo et al. 2021), further suggesting a loss-of-function mechanism. It should be noted that pathochmachnisms independent from *RAC1* activation are also conceivable. For example, Ueda et al. (2013) demonstrated that proline-rich C terminus of *DOCK4* is required for dendritic spine formation via interaction with cortactin. Furthermore, *DOCK4* is the only member of the DOCK family that is capable of activating RAP1 (Yajnik et al. 2003; Shi 2013). Therefore, different underlying pathomechanisms and thus a genotype–phenotype correlation for *DOCK4*-related NDD seem plausible and need to be investigated in future studies.

A limitation of the study is the unavailability of comprehensive phenotypic information for each individual. Additionally, the use of a murine neuroblastoma cell line (Neuro-2A) to study the effect of variants identified in humans is also a limitation. Although we used the human *DOCK4* sequence, which is highly conserved (98.18%% protein sequence similarity to murine Dock4), for our overexpression studies, the genetic background is indeed different. The use of common human cell lines such as SHSY5Y would be beneficial in this regard, but this cell line is also derived from neuroblastoma and is therefore also a limitation to investigate NDD. Nevertheless, this approach is still being used successfully in recent studies to validate the impact of missense variants associated with human disorders (Cioclu et al. 2023). Another limitation of the present study is the analysis of bi-allelic *Dock4* KO cells, while individuals 5–7 carry heterozygous null variants. We have chosen bi-allelic *Dock4* KO cells, as relevant effects could be concealed by the analysis of differentiated cells, compared to effects that take place mostly during early development, as demonstrated in heterozygous *Cux*^**+/−**^ mice (Oppermann et al. 2023). However, novel approaches such as CRISPR/Cas-based methods, the use of human-induced pluripotent stem cells and single-cell analysis will overcome these limitations in the future (Kurishev et al. 2022; Huang et al. 2023).

In summary, the overlapping phenotype of seven Individuals, the structural modeling, the role of *DOCK4* in the central nervous system and the proven impact of the variants on neuronal outgrowth prompt us to add heterozygous null variants and deleterious missense variants in *DOCK4* as a probable monogenetic cause of an NDD with microcephaly. However, replication with larger cohorts is necessary.

### Internet Resources

GeneMatcher, https://genematcher.org/

gnomAD, https://gnomAD.broadinstitute.org/

MetaDome, https://stuart.radboudumc.nl/metadome

OMIM, https://omim.org/

The National Center for Biotechnology Information (NCBI), https://www.ncbi.nlm.nih.gov/

UCSC Cell Browser (human cerebral cortex), https://cells.ucsc.edu/

Variant Effect Predictor (VEP) from ENSEMBL, https://www.ensembl.org/

DECIPHER, https://decipher.sanger.ac.uk/

GenBank, https://www.ncbi.nlm.nih.gov/genbank/

UniProt database, https://www.uniprot.org/

STRING database, https://www.string-db.org/

WebAutoCasC, https://autocasc.uni-leipzig.de/

### Supplementary Information

Below is the link to the electronic supplementary material.

Supplementary file1 (PDF 1327 kb)

Supplementary file2 (XLSX 25 kb)

## Data Availability

The data of this study are available from the corresponding author within reasonable request. There was no code used for this study.
