# Supplementary material for: Heterozygous loss-of-function variants in DOCK4 cause neurodevelopmental delay and microcephaly
Source: Hum Genet. 2024 Mar 25;143(3):455–69. doi: 10.1007/s00439-024-02655-4 (PMC11043173; doi:10.1007/s00439-024-02655-4)
Supplement: Supplementary file 1 — Supplementary file1 (PDF 1327 kb) [file 439_2024_2655_MOESM1_ESM.pdf]

## SUPPLEMENTAL DATA

## SUPPLEMENTAL INFORMATION: CASE REPORTS

**Family 1:**The male individual was born in the 40th gestational week after uneventful pregnancy spontaneously to healthy nonconsanguineous parents. Till the age of 4 years the development was appropriate. The tonic clonic seizures started at the age of 4 years. From this point the neurodevelopmental delay started. At the age of 16 years he had pharmacoresistant focal to bilateral tonic-clonic seizures. A MRI at the age of 15 years was normal, without any signs of cerebellar abnormality. He presented tremor, bilateral kidney cysts and an intellectual disability. A formal testing is not possible, but based on a clinically evaluation the intellectual disability can be graduated as moderate to severe (developmental aged delayed for more than 6 years in comparison to age-matched peers).

**Family 2:** The male individual was born in the 39th gestational week (birth weight= 2930g, Z-score -1.28; length = 50 cm, Z-score -0.83; OFC= 32.5 cm, Z-score -2.15) to non-consanguineous parents. At the age of 5 years he presented with a neurodevelopmental delay and intellectual disability. An MRI at the age of 18 months showed mild cerebellar ectopia. He presented at age 18 months with ataxia, hypotonia, spasticity in the legs and a microcephaly (OFC= 43.3 cm, Z-score -3.84). He showed wide/low nasal bridge, full cheeks, narrow forehead and amblyopia.  The patient's father, who transmitted the variant c.758C>T, p.Pro253Leu, has a history of bipolar, ADHD, anxiety, dyslexia and special education services in school.

**Family 3**: The male individual was born at 40 + 4th gestational week (birth weight= 3350g, Z-score -0.76; length = 50 cm, Z-score -1.24) to healthy non-consanguineous parents with no antenatal or neonatal complications. Apgar scores were 1 min: 9; 5min: 9. His paternal grandmother was diagnosed with a Wolf Parkinson's White Syndrome. There was no other significant family history. He presented at the age of 9 months with a neurodevelopmental delay, showing slow progress in his gross and fine motor skills, and seeming to keep his right thumb tucked in. On examination, right hemiplegia with fixed plantar flexure contracture of right ankle was noted. He has increased tone in his right calf which can only be stretched to neutral position. There is spasticity present, as well as signs of dystonia in the right upper limb with variable tone and posture and right thumb is mostly opposed and adducted. He has some tightness in pronators. He was diagnosed with right hemiplegic cerebral palsy with dystonia. MRI showed at the age of 18 months volume loss in left periventricular region, extending into basal ganglia and even part of brainstem, with thinning of the corpus callosum and suggestion of incomplete myelination in surrounding areas. The left lateral ventricle showed compensatory dilation. At 18 months, he walks independently but often trips and drags his right foot. He used two hands in play, but tended to flex his right arm in an intermittent fashion, and had become left hand dominant. He expressed several words and babbled and points in communication and on examination showed near age appropriate communication skills. At 3 years, his developmental was reported to be within the normal range. He had GMFCS 2 MACS 1 at 6 years of age and stable onwards. There are no other health concerns.

**Family 4:** The male individual was born to non-consanguineous parents and was adopted at the age of 5 years. Except for mild cerebral palsy, no clinical information was known about the period prior to adoption. Clinical assessment revealed a severe neurodevelopmental delay,

intellectual disability (moderate), deficits in social-emotional reciprocity, poor eye contact and an attention deficit hyperactivity disorder. He has generalized hypotonia, dystonia, periodic episodes of unilateral extremity weakness, lethargy, astigmatism and myopia. An EEG indicated focal cerebral dysfunction and complex partial seizures. Craniofacial dysmorphic features include mild brachycephaly, relative macrocephaly, hypertelorism, narrow arched eyebrows, upward slanting palpebral fissures and a depressed nasal bridge. An MRI showed left posterolateral positional plagiocephaly, enlarged bilateral subarachnoid spaces, sulci, and borderline-enlarged lateral and third ventricles, most consistent with mild diffuse cerebral atrophy, minimal nonspecific white matter gliosis (minimal amount of gliosis might be considered within normal limits for patient age).

**Family 5:** The male individual was born in the 38+1 gestational week with birth weight 2680g to healthy non-consanguineous parents. At age 3.5 years, the kindergarten teacher noticed he has difficulties concentrating, and he was referred for further evaluation. At age 6 years he is diagnosed with global developmental delay, intellectual disability (language DQ score 61, performance DQ score 56) and autism spectrum disorder. He presents with microcephaly (OFC 48 cm, Z-score -2) but normal height (111.5 cm, 68th percentile) and weight (19.6 kg, 65th percentile), thick eyebrows, mild fifth finger clinodactyly, and abnormal blinking. A brain MRI performed at the age of 5 years was normal. In addition, he is diagnosed with familial ichthyosis due to a known Xp22.31 microdeletion, which includes the *STS* gene, identified by both microarray and exome sequencing.

**Family 6:** The female individual was born (birth weight= 3560g, Z-score -0.21; length = 51,5 cm, Z-score -0.1; OFC= 35 cm, Z-score -0.1) as the second child of healthy, non-consanguineous parents. She had five half-brothers and sisters and two of them had mild learning difficulties. She presented at the age of 20 years with a mild neurodevelopmental delay, global learning difficulties and an attention disorder. Moreover, she presented with anxiety. Further symptoms were strabismus and glossodynia with "geographic tongue". Clinical examination was normal but we could note micrognathia. The mother had learning difficulties, reading difficulties and attention disorder. The sister had mild psychomotor delay (walking at 2 years, speech delay) and learning difficulties, reading difficulties, memory deficit, anxiety, stable tremor without cerebellar involvement. Other family maternally members have learning difficulties. The variant c.2770C>T, p.Arg924* in *DOCK4* was detected in the index heterozygous, but not in the mother and not in the sister. There is no contact to the father

**Family 7:** The male individual was born at 32 gestational weeks (birth weight= 2450g, Z-score 1.14; length = 48,5 cm, Z-score 1.46; OFC= 33 cm, Z-score 1.13) to a non-consanguineous couple; prenatal ultrasounds were normal but mother noted decreased fetal movements during the pregnancy. At 3 days of life he had a right lung collapse requiring chest tube placement. At 11 months of age a neurology evaluation demonstrated hypotonia and tongue fasciculations, leading to testing for spinal muscular atrophy which was negative. Further symptoms included esotropia and inverted nipples, which led to screening for congenital disorders of glycosylation. He continued to have generalize hypotonia, and a brain MRI at 10 months showed modest prominence of the CSF spaces with appearance of thinning of the corpus callosum and white matter in the centrum semiovale. He later developed more axial

hypertonia with persistent central hypotonia and was diagnosed with cerebral palsy. Repeat MRI at 21 months showed bilateral cerebral hemispheric white matter volume loss and gliosis. At age 3 he had bilateral media rectus recession for esotropia. At 4 years there were concern for spells, with 20-30 episodes a day (occurring in clusters of 5-6) of arm extension and head deviation; EEG showed right posterior spikes suggestive of localization related epilepsy and he was prescribed clobazam.

Developmentally, he showed a moderate developmental delay. At the age of 14 months, his therapy team evaluated his physical development to be that of a 6 months, communication at 8 months, self-care at 10 months, and social skills at 9 months equivalence. At the age of 20 months he could roll but not bear weight on his legs, would repeat words like “no” and “nap,” and have head-banging episodes. He was speaking by age 3 years. By 3 years of age he could walk with a walker and at age 5 years is in the process of getting a gait-trainer that would be self-propelled with pushing. Also at 5 years he can briefly stand without assistance, scribble, feed himself, and use utensils. He is not toilet trained.

At the age of 4 years and 11 months he was 96 cm tall (Z-score -2.65) and weighted 14.7 kg (Z-score -1.86, BMI: 15.95). The OFC at the age of 1 year and 8 months was 46 cm (Z-score -1.19).

**Family 8:** The male individual was born premature at 28+4th gestational week. He presented at 47 months of age with severe global developmental delay, incomplete head control, incomplete roll over and no sitting or grasping. Moreover, the individual presented with microcephaly and encephalopathy. No seizures were observed so far and the MRI was within

the normal range. Microarray, Fragile- X screening and metabolic screening tests were all negative.

**Family F1:** A 28-year-old woman, gravida II, para I, underwent routine ultrasonography of the second trimester at 23 weeks of gestation (WG) which revealed microcephaly associated to an enlarged posterior fossa with small cerebellum and probable vermian agenesis. Based on these findings, a medical termination of the pregnancy (TOP) was achieved at 24 WG in accordance with the French law. The parents were not consanguineous and medical history of the parents was unremarkable. Blood markers for trisomy 21 were normal, and chromosomal analysis performed on amniotic fluid cells revealed a normal male karyotype, 46, XY.

A complete autopsy was performed with the informed consent of both parents in accordance with French law and following standardized protocols. Growth parameters were normal as well as skeletal measurements (length 30 cm, 50$^{th}$ percentile). The main distinct facial features, though not specific, included broad nasal ridge, short nose with bulbous tip, short philtrum and retrognathism. Cranial perimeter was <3$^{rd}$ percentile (measured at 19cm; N 24 WG =23.3 cm) (Guihard-Costa and Larroche 1990; Guihard-Costa et al. 2002) indicative of microcephaly. Visceral examination displayed neither macroscopic nor histological anomalies.

On macroscopic brain examination the brain weight was below the 3$^{rd}$ percentile (14g instead of 112g at 25 WG) as were weights and cerebellar transverse diameters (15 mm instead of 26.1 mm at 25 WG) corresponding to a developmental age of 15 WG (Garel 2000). On external examination, no primary fissures were observed. Conversely, olfactory bulbs and optic chiasm were present, and on supratentorial coronal sections the corpus callosum was identified, and

there was no dilatation of the lateral and 3rd ventricles. On sections passing through the mesencephalon, the aqueduct of Sylvius and the cerebral peduncles were identified. The 4th ventricle was enlarged and the cerebellum was severely hypoplastic, forming a single and non-foliated mass, arguing for a rhombencephalosynapsis with severe maturation delay. Histologically, the brain displayed exceedingly severe lesions located in the brainstem and cerebellum. The dentate nuclei as well as the inferior olivary nuclei were missing and pontine grey nuclei and transverse pontine fibres were strongly hypoplastic, as were the pyramidal tracts. No acquired lesions were observed in any of the different supratentorial brain structures. In the cortical plate neuronal density was severely decreased. The deep grey nuclei were rudimentary, but the corpus callosum was normal and no migrational anomalies were observed in the intermediate zone. The spinal cord and the eyes were also histologically normal.

**_DOCK4_ variants detected in individuals with a neurodevelopmental disorder from the literature**

Kaplanis *et al.* (Kaplanis et al. 2020) analyzed 9858 trios from 9307 families from patients with severe, undiagnosed developmental disorders. Among them were four individuals with *de novo* variants in *DOCK4* (ID: GDX_1030, c.395G>A, p.Gly132Asp; ID: GDX_54555, c.4135+1G>T, p.?; ID:; GDX_67215, c.2035A>G, p.Ile679Val; ID: rumc_patient_2014, c.4145T>A, p.Phe1373Tyr). In tis study, the *de novo* *DOCK4* variants were not statistically enriched (p=0,136). Iossifov *et al.* (Iossifov et al. 2014) analyzed 2630 families with intelectual disability. In one male individual with borderline ID (verbal IQ:77, non-verbal IQ: 89) the *de novo* variant c.4250G>A, p.Arg1417His was detected. Homsy *et al.* (Homsy et al. 2015), analyzed 1213 trios from individuals with congenital heart disease and a neurodevelopmental disorder and identified one individual with a neurodevelopmental disorder, hypoplastic left heart syndrome, mitral atresia, aortic atresia and the *de novo* variant c.1382A>G, p.His461Arg. Pagnamenta *et al.* (Pagnamenta et al. 2010) described a family with dyslexia in which eight individuals (two females

and six males) harbor a *DOCK4* truncating deletion (p.Asp946_Lys1966delinsValSer*). One mother (index) with an unremarkable development and dyslexia had two sons and one daugther. The daughter, like the mother, had an unremarkable development and dyslexia. The two sons had DD and were diagnosed with autism. The IQ was in the normal range. One brother of the mother was also diagnosed with dyslexia (developmental milestones are unknown). He had two sons with the deletion who had significant problems in reading and spelling (developmental milestones are unknown). A second brother of the mother was diagnosed with Asperger disorder (developmental milestones are unknown). It should be noted that the brothers of the mother (index) also inherited a deletion from their father that disrupts *CNTNAP5*. The father has average reading ability and higher education. Although the father appears to be clinically unremarkable, Aleo *et al.* (Aleo et al. 2020) reported two individuals with a deletion affecting *CNTNAP5* and reduced IQ and DD. One had autism. As a gene-disease association for *CNTNAP5* has not been confirmed, yet, it is unknown how the *CNTNAP5* deletion contributed to the phenotype of the brothers. Pagnamenta *et al.* concluded that the *DOCK4* deletion is possibly associated with dyslexia, as this deletion co-segregates in the family.

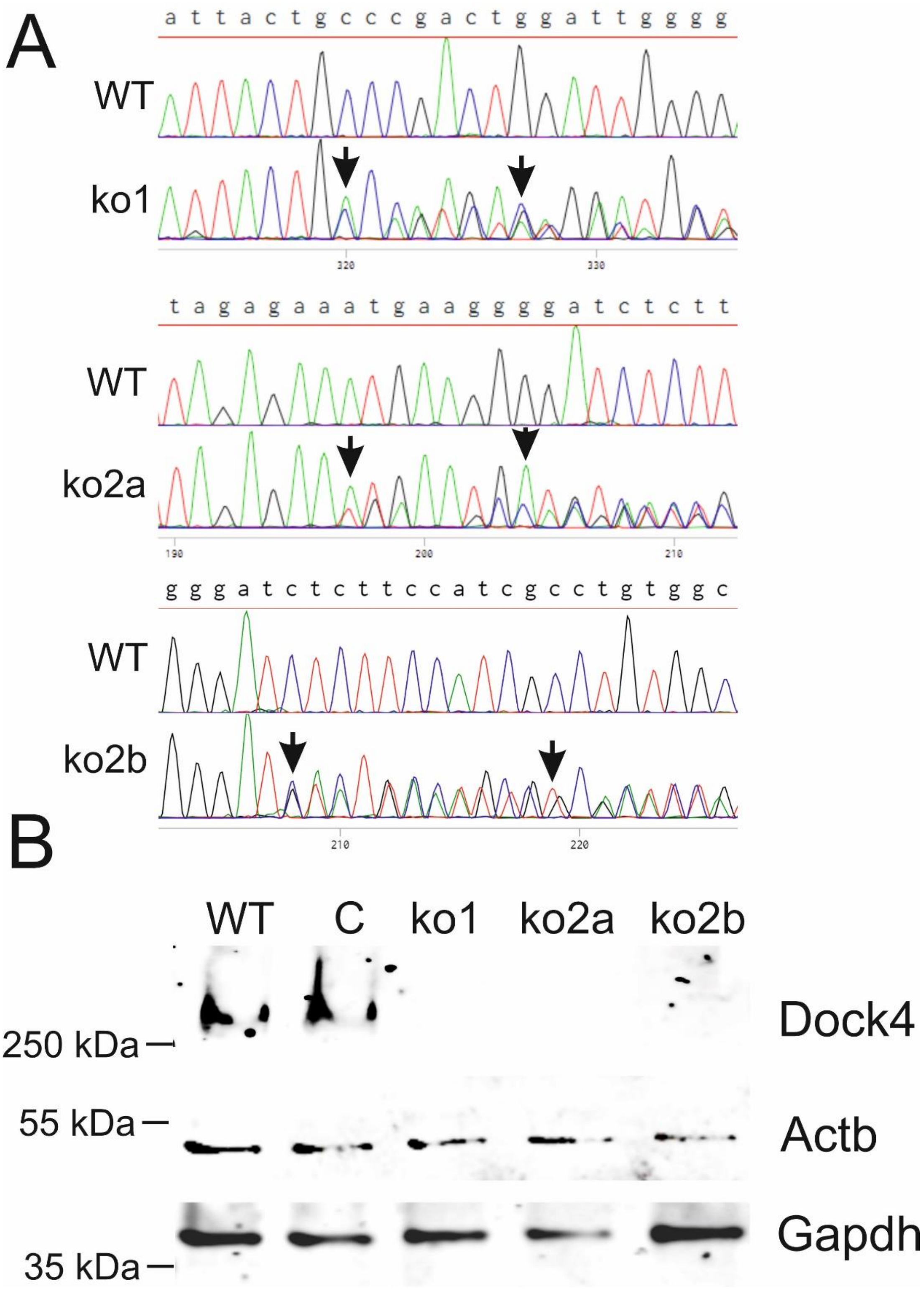


**Figure S1: *Dock4* knout-out in Neuro-2A cells.** (A) DNA sequencing of wild-type (WT), ko1 and ko2 Neuo-2A cells. Arrows indicate the start of each allelic frameshift. (B) Western Blot of WT, control (C) ko1 and ko2 Neuo-2A cells confirming *Dock4* knock-out Actb and Gapdh were used as loading controls.

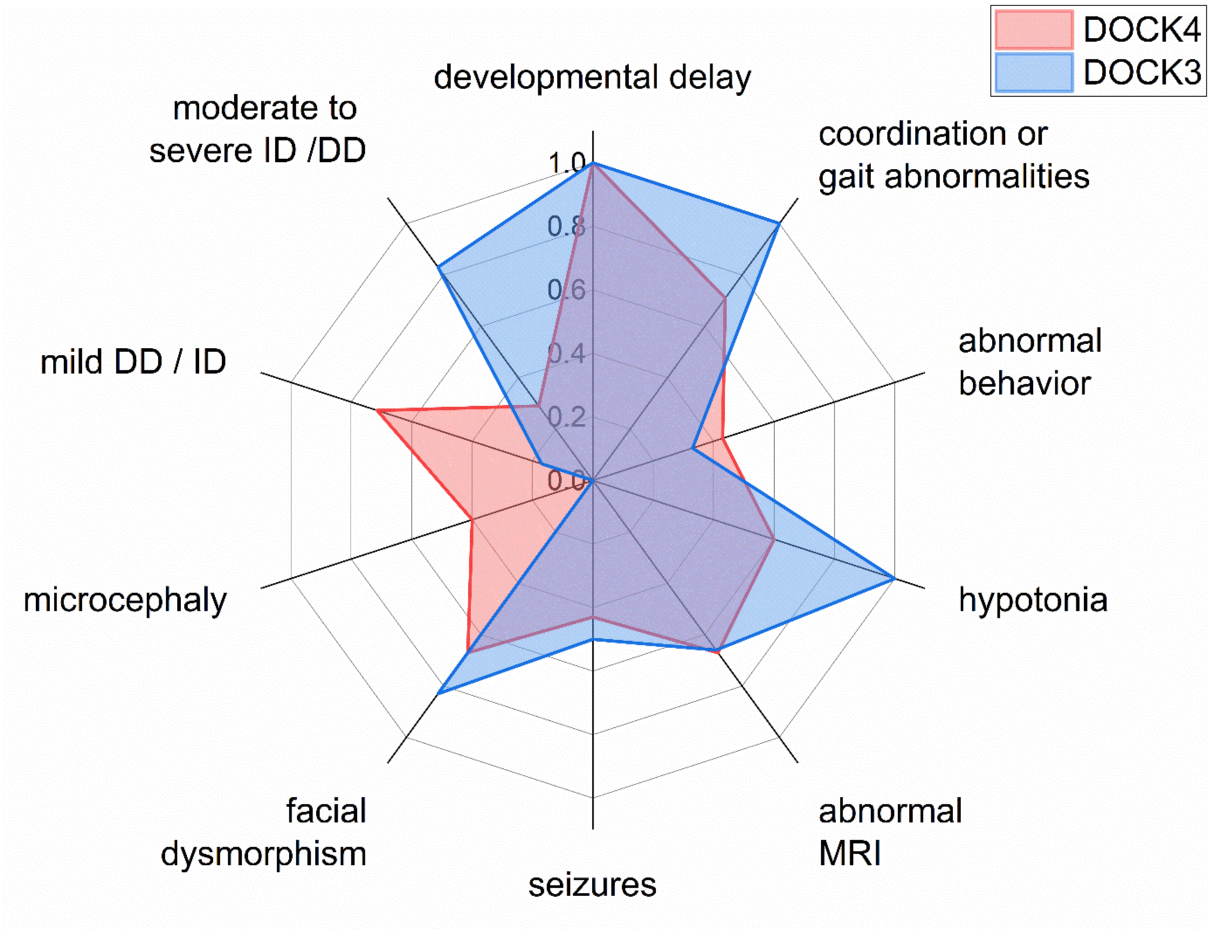


**Figure S2: Phenotypic overlap of *DOCK3* and *DOCK4* Individuals:** Proportion of individuals with certain symptoms with variants in *DOCK3* (Wiltrout et al. 2019) (blue) or *DOCK4* (red) are illustrated as radar plot. Abbreviation: DD: global developmental delay; ID: intellectual disability; MRI: magnetic resonance imaging

## References

Garel C (2000) Le développement du cerveau foetal. Atlas IRM et biométrie. Sauramps médical, Montpellier

Guihard-Costa AM, Larroche JC (1990) Differential growth between the fetal brain and its infratentorial part. Early human development 23(1):27–40

Guihard-Costa A-M, Ménez F, Delezoide A-L (2002) Organ weights in human fetuses after formalin fixation: standards by gestational age and body weight. Pediatric and developmental pathology : the official journal of the Society for Pediatric Pathology and the Paediatric Pathology Society 5(6):559–578

Homsy J, Zaidi S, Shen Y, Ware JS, Samocha KE, Karczewski KJ, DePalma SR, McKean D, Wakimoto H, Gorham J, Jin SC, Deanfield J, Giardini A, Porter GA, Kim R, Bilguvar K, López-Giráldez F, Tikhonova I, Mane S, Romano-Adesman A, Qi H, Vardarajan B, Ma L, Daly M, Roberts AE, Russell MW, Mital S, Newburger JW, Gaynor JW, Breitbart RE, Iossifov I, Ronemus M, Sanders SJ, Kaltman JR, Seidman JG, Brueckner M, Gelb BD, Goldmuntz E, Lifton RP, Seidman CE, Chung WK (2015) De novo mutations in congenital heart disease with neurodevelopmental and other congenital anomalies. Science (New York, N.Y.) 350(6265):1262–1266

Iossifov I, O'Roak BJ, Sanders SJ, Ronemus M, Krumm N, Levy D, Stessman HA, Witherspoon KT, Vives L, Patterson KE, Smith JD, Paeper B, Nickerson DA, Dea J, Dong S, Gonzalez LE, Mandell JD, Mane SM, Murtha MT, Sullivan CA, Walker MF, Waqar Z, Wei L, Willsey AJ, Yamrom B, Lee Y, Grabowska E, Dalkic E, Wang Z, Marks S, Andrews P, Leotta A, Kendall J, Hakker I, Rosenbaum J, Ma B, Rodgers L, Troge J, Narzisi G, Yoon S, Schatz MC, Ye K,

McCombie WR, Shendure J, Eichler EE, State MW, Wigler M (2014) The contribution of de novo coding mutations to autism spectrum disorder. Nature 515(7526):216–221

Kaplanis J, Samocha KE, Wiel L, Zhang Z, Arvai KJ, Eberhardt RY, Gallone G, Lelieveld SH, Martin HC, McRae JF, Short PJ, Torene RI, Boer E de, Danecek P, Gardner EJ, Huang N, Lord J, Martincorena I, Pfundt R, Reijnders MRF, Yeung A, Yntema HG, Vissers LELM, Juusola J, Wright CF, Brunner HG, Firth HV, FitzPatrick DR, Barrett JC, Hurles ME, Gilissen C, Retterer K (2020) Evidence for 28 genetic disorders discovered by combining healthcare and research data. Nature 586(7831):757–762

Pagnamenta AT, Bacchelli E, Jonge MV de, Mirza G, Scerri TS, Minopoli F, Chiocchetti A, Ludwig KU, Hoffmann P, Paracchini S, Lowy E, Harold DH, Chapman JA, Klauck SM, Poustka F, Houben RH, Staal WG, Ophoff RA, O'Donovan MC, Williams J, Nöthen MM, Schulte-Körne G, Deloukas P, Ragoussis J, Bailey AJ, Maestrini E, Monaco AP (2010) Characterization of a family with rare deletions in CNTNAP5 and DOCK4 suggests novel risk loci for autism and dyslexia. Biological psychiatry 68(4):320–328

Wiltrout K, Ferrer A, van de Laar I, Namekata K, Harada T, Klee EW, Zimmerman MT, Cousin MA, Kempainen JL, Babovic-Vuksanovic D, van Slegtenhorst MA, Aarts-Tesselaar CD, Schnur RE, Andrews M, Shinawi M (2019) Variants in DOCK3 cause developmental delay and hypotonia. European Journal of Human Genetics 27(8):1225–1234
